# Opposing effects of *in vitro* differentiated macrophages sub-type on epithelial wound healing

**DOI:** 10.1371/journal.pone.0184386

**Published:** 2017-09-01

**Authors:** Julia A. Gindele, Samuel Mang, Nicolas Pairet, Ingrid Christ, Florian Gantner, Jürgen Schymeinsky, David J. Lamb

**Affiliations:** 1 Immunology & Respiratory Diseases Research, Boehringer Ingelheim Pharma GmbH & Co. KG, Biberach an der Riß, Germany; 2 Department of Biology, University of Konstanz, Konstanz, Germany; 3 Institute of Immunology, Hannover Medical School, Hannover, Germany; 4 Department of General Physiology, University of Ulm, Ulm, Germany; 5 Translational Medicine and Clinical Pharmacology, C. H. Boehringer Sohn AG & Co. KG, Biberach an der Riß, Germany; University of Alabama at Birmingham, UNITED STATES

## Abstract

Inappropriate repair responses to pulmonary epithelial injury have been linked to perturbation of epithelial barrier function and airway remodelling in a number of respiratory diseases, including chronic obstructive pulmonary disease and idiopathic pulmonary fibrosis. We developed an *in vitro* mechanical scratch injury model in air-liquid interface differentiated primary human small airway epithelial cells that recapitulates many of the characteristics observed during epithelial wound injury in both human tissue and small animal models. Wound closure was initially associated with de-differentiation of the differentiated apical cells and rapid migration into the wound site, followed by proliferation of apical cells behind the wound edge, together with increases in FAK expression, fibronectin and reduction in PAI-1 which collectively facilitate cell motility and extracellular matrix deposition. Macrophages are intimately involved in wound repair so we sought to investigate the role of macrophage sub-types on this process in a novel primary human co-culture model. M_1_ macrophages promoted FAK expression and both M_1_ and M_2_ macrophages promoted epithelial de-differentiation. Interestingly, M_2a_ macrophages inhibited both proliferation and fibronectin expression, possibly via the retinoic acid pathway, whereas M_2b_ and M_2c_ macrophages prevented fibronectin deposition, possibly via MMP expression. Collectively these data highlight the complex nature of epithelial wound closure, the differential impact of macrophage sub-types on this process, and the heterogenic and non-delineated function of these macrophages.

## Introduction

Inappropriate repair responses to macro- and micro-pulmonary epithelial injury have been linked to perturbation of epithelial barrier function and airway remodelling, possibly as a consequence of epithelial-mesenchymal transition, in a number of respiratory diseases, including chronic obstructive pulmonary disease (COPD) [1] and idiopathic pulmonary fibrosis (IPF) [2]. Whilst the precise mechanisms that are evoked during epithelial wound healing remain controversial [3], it is believed that cells at the wound edge dedifferentiate and flatten enabling migration over the damaged area. The epithelial basal cells then proliferate and the wound becomes filled with undifferentiated cells [4]. Subsequently, it is believed that a process of de-differentiation takes place and extracellular matrix proteins such as fibronectin are produced, which form the scaffold for the regenerated epithelium [5–7] although it may take several days before the injury is fully repaired [4].

Tissue macrophages are often described as existing in polarised states often termed M_1_ and M_2_, although recent data suggest that such cells exist in a more heterogeneous state [8]. In IPF, a number of groups have described lung macrophages as possessing M_2_-associated phenotypes, such as CD163 and it has been discussed that such macrophages are associated with a tissue remodelling phenotype. In contrast, macrophages have been associated with COPD exacerbations [9] and that the inflammatory response may promote an M_1_ phenotype. Indeed, granulocyte-macrophage colony stimulating factor (GM-CSF), an M_1_ macrophage differentiation factor, and product of cells activated during inflammation, are elevated during COPD [10, 11].

In this study, we sought to model and characterise the pulmonary epithelial response to injury using mechanical scratching of primary human small airway epithelial cells in air-liquid interface culture as a test system. Furthermore we aimed to explore the role of primary human macrophages differentiated *ex vivo* into different phenotypes on this process in a novel primary human cell co-culture system.

## Results

### Characterisation of epithelial injury

**Fig 1:**
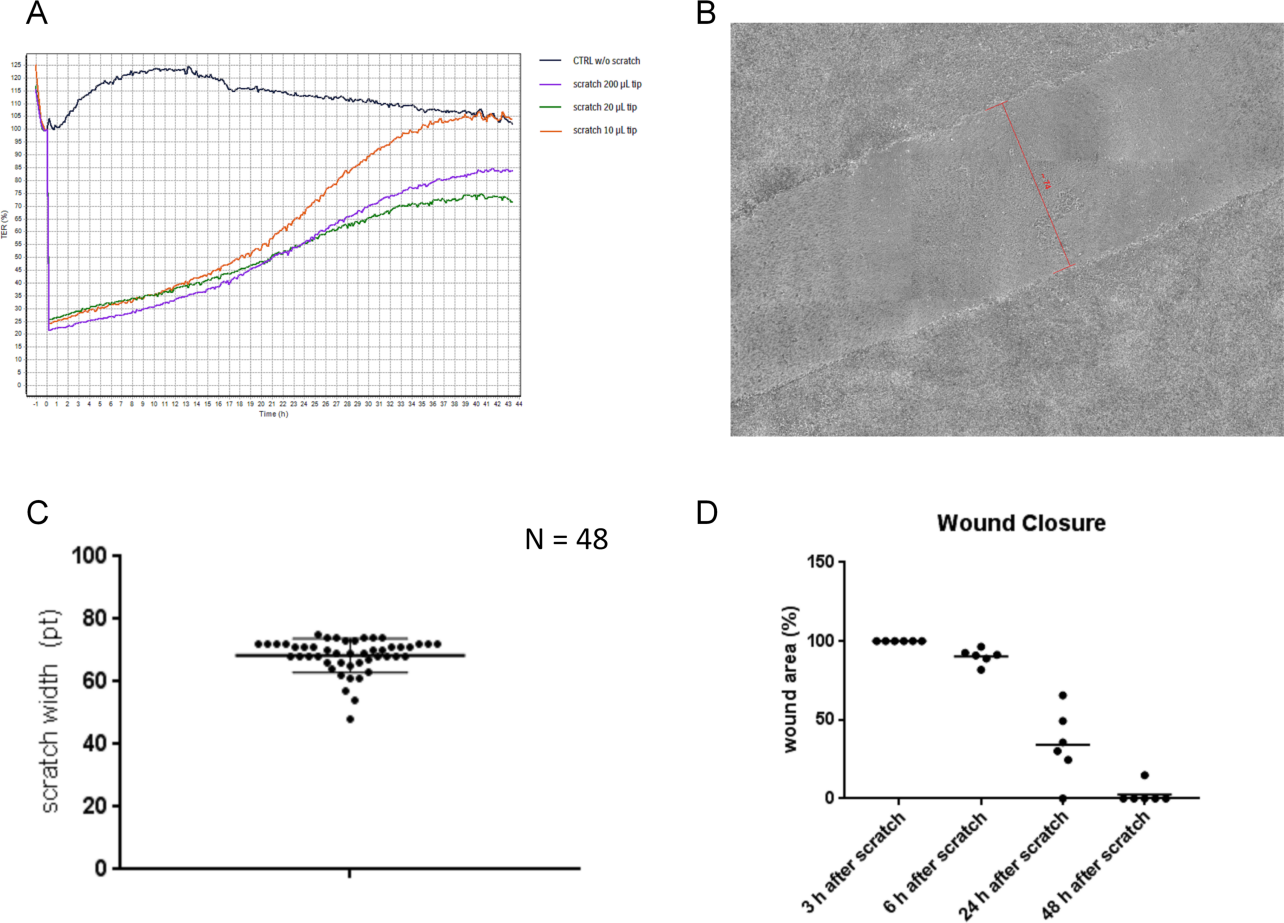
**TEER development and Scratch width measurement. A** TEER development measured in the cellZScope over time using no (black line), a 200 μL (orange line), a 20 μL (green line) and a 10 μL (violet line) pipet tip. **B** Microscopic image (CellIQ) of a scratch introduced with a 200 μL pipette tip (width (74 pts) of the scratch indicated by the red line) **C** scratch width (mean width 68.38 ± 5.39 pts) measured and plotted to demonstrate reproducibility of the scratch (n = 48) **D** time course of the closure of the scratch (n = 6)

The air-liquid interface differentiated human small airway epithelial cell culture inserts were injured by scratching the surface with 10 μL, 20 μL or 200 μL plastic pipette tip and monitoring the transepithelial electrical resistance (TEER; Fig 1A). TEER completely recovered within 40 hours of scratching with a 10 μL pipette tip, but recovered to only 70–80% control after 44 hours of scratching with a 20 μL or 200 μL pipette tip. A 200 μL pipette tip was chosen for subsequent experiments, because it produced a uniform scratch comprising approximately 20% of the total cell area. The mean width of the scratch was measured using the CellIQ (Fig 1B). In 48 consecutive scratches, the mean width was 68.4 ± 5.4 pts (Fig 1C). Next, we measured the dynamics of closure of the scratch by measuring the scratch size at different time points. The width of the scratch was reduced at 6 hours, 24 hours and 48 hours by 10%, 66% and 97% respectively (Fig 1D). The response to injury appeared to be initiated from a series of “hot spots” in cell clusters adjacent to the wound edge, rather than a homogenous response along the wound edge (S1 Video). We observed that, if the underlying collagen coating was damaged during the scratch (lower scratch border, S2 Video) then the response to injury was impaired along the site of damage until wound closure was almost complete.

**Fig 2:**
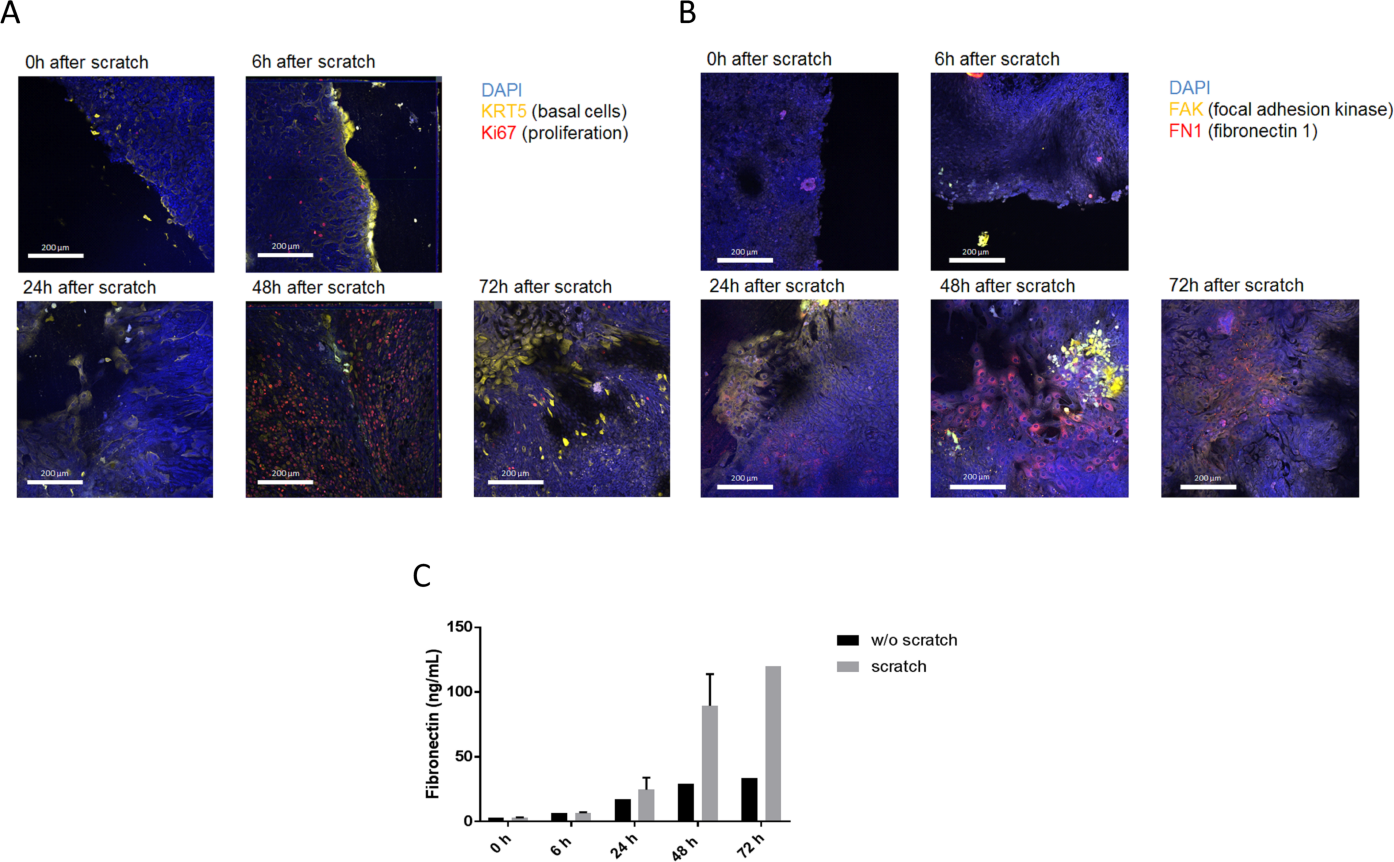
**IHC of scratched cells and fibronectin expression. A** IHC microscopic images of scratched cells at t = 0h, 6h, 24h, 48h and 72h; DAPI in blue, KRT5 in yellow, Ki67 in red. **B** IHC microscopic images of scratched cells at t = 0h, 6h, 24h, 48h and 72h; DAPI in blue, FAK in yellow, FN1 in red. **C** ELISA of FN1 in the supernatant after 0h, 6h, 24h, 48h and 72h with and without scratch.

Immunocytochemistry staining reveals KRT5-positive basal cells are prominent at the leading edge of wound repair 6 hours after injury, but thereafter are not seen at the leading edge (Fig 2A). Wound closure between 24 and 72 hours appears to be associated with a reduction in expression of both tight junction associated molecules (CLDN3, OCLN) and differentiation markers for ciliated cells (FOXJ1), club cells (SCGB1A1), columnar epithelial cells (TFF3), and α-smooth muscle actin (ACTA2) (Table 1).

**Table 1:** Regulation of selected genes in small airway epithelial cells after injury.

Molecule association	Gene	Fold regulation in expression after injury			
		6h	24h	48h	72h
Tight junctions	CLDN3	1,29	-1,98	-2,54	-1,25
	OCLN	2,08	-1,50	-1,55	-1,19
Differentiation	FOXJ1	1,69	-1,77	-1,38	-1,60
	SCGB1A1	1,79	-3,66	-2,69	-2,79
	TFF3	1,16	-1,78	-2,36	-1,97
	ACTA2	1,60	-1,09	-1,27	-1,30
Proliferation	Ki67	1,24	1,44	6,26	10,81
Extracellular matrix	FN1	-1,74	-1,80	1,25	2,72

Interestingly, Ki67-positive proliferating cells are observed behind the leading edge from 6 hours onwards (Fig 2A) and the increase in cellular proliferation was confirmed by a significant increase in Ki67 mRNA observed 48 and 72 hours after injury (Table 1). Both focal adhesion kinase (FAK) and fibronectin staining was present on the leading edge of the 24 and 48 hours after injury and fibronectin (FN1) was still present 72 hours after injury when the wound had been physically closed (Fig 2B). This was accompanied by an increase in soluble fibronectin 48 and 72 hours after injury (Fig 2C).

### Characterisation of macrophage phenotype for co-culture experiments

Monocyte-derived macrophages were differentiated into “M_1_” and “M_2_” phenotypes by culturing with GM-CSF and M-CSF respectively for 7 days. “M_2_” macrophages were incubated with IL-4/IL-13, immune complexes/LPS, or IL-10 to generate “M_2a_”, “M_2b_” and “M_2c_” sub-phenotypes. CD68 was uniformly expressed across the different sub-types, whereas CD80 was expressed on at low levels on all macrophages sub-types. CD163 was predominantly expressed on “M_2c_” macrophages.

### Effect of macrophage phenotype on epithelial injury response

**Fig 3:**
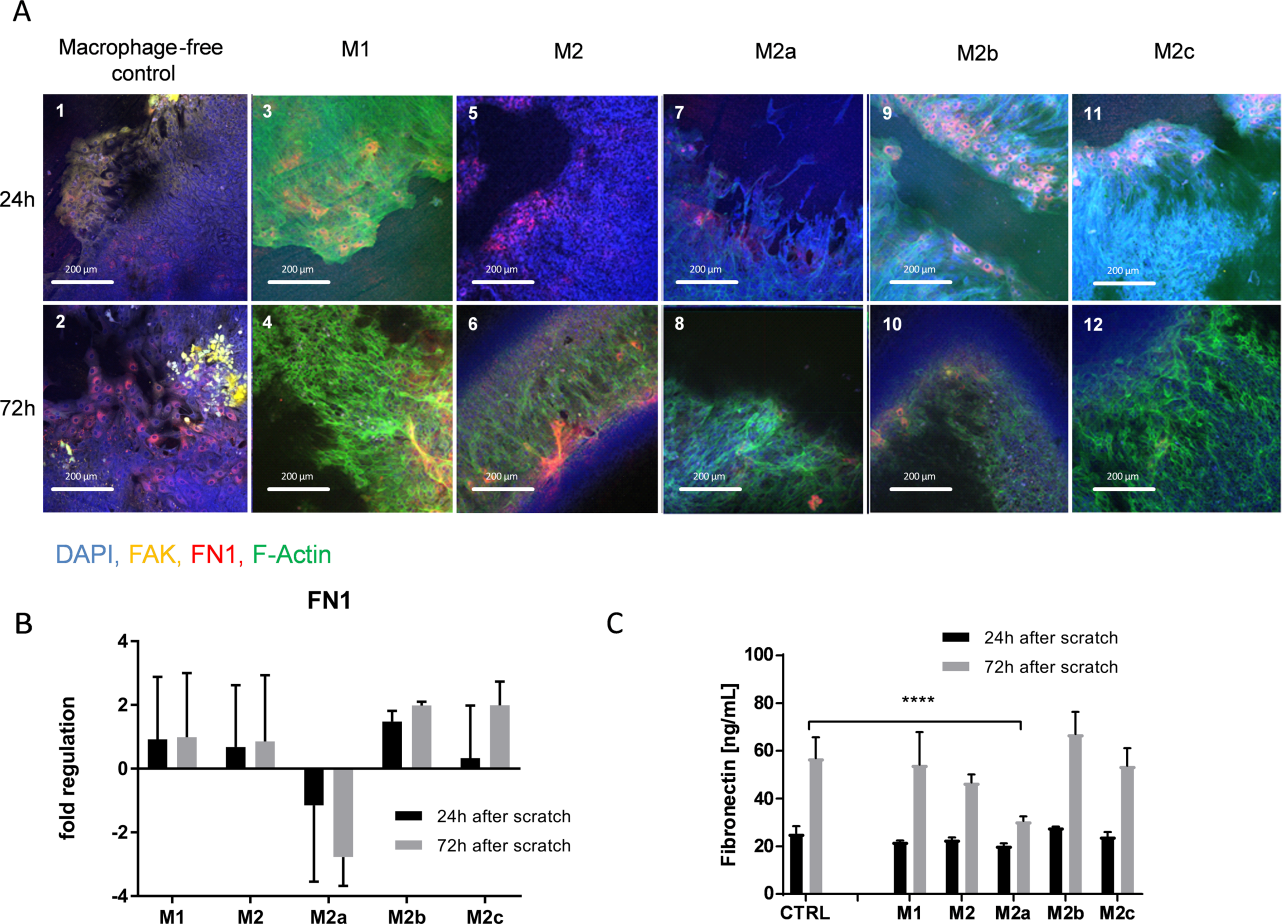
**IHC of scratched cells and FN1 regulation/expression. A** scratched epithelial cells incubated with different macrophage subtypes after 24h and 72h; DAPI stained in blue, FAK stained in yellow, FN1 stained in red, F-Actin stained in green. **B** FN1 mRNA regulation in the co-culture cell lysates measured after 24h and 72h Data are displayed as fold regulation compared to control medium containing macrophage maturation factors; Data is expressed as mean ± SD; n = 3 **C** FN1 expression measured in the supernatants via ELISA after 24h and 72h; Data is expressed as mean ± SD; n = 3 (**** = p>0.0001)

Addition of non-stimulated “M_1_” macrophages increased F-actin staining in epithelial cells at both 24 and 72 hours post-injury (Fig 3A, panels 3 and 4). “M_2_” macrophages also increased F-actin staining, but only 72 hours post-injury (Fig 3A, panel 6). “M_2_” macrophages also suppressed FAK staining both 24 and 72 hours post-injury (Fig 3A, panels 5 and 6).

Fibronectin staining in the epithelium after injury was also reduced by “M_2a_” macrophages 24 and 72 hours after injury (Fig 3A, panels 7 and 8). Interestingly, intracellular fibronectin was observed 24 hours after injury in epithelial cells co-cultured with “M_2b_” and “M_2c_” macrophages, but was not apparent 72 hours after injury (Fig 3A, panels 9–12). Epithelial fibronectin gene expression was reduced by the “M_2a_” macrophage subtype, but not with “M_2b_” or “M_2c_” subtypes (Fig 3B) and soluble fibronectin was also decreased in the co-cultures with “M_2a_” but not “M_2b_” or “M_2c_” cultures 72 hours after injury (Fig 3C).

**Fig 4:**
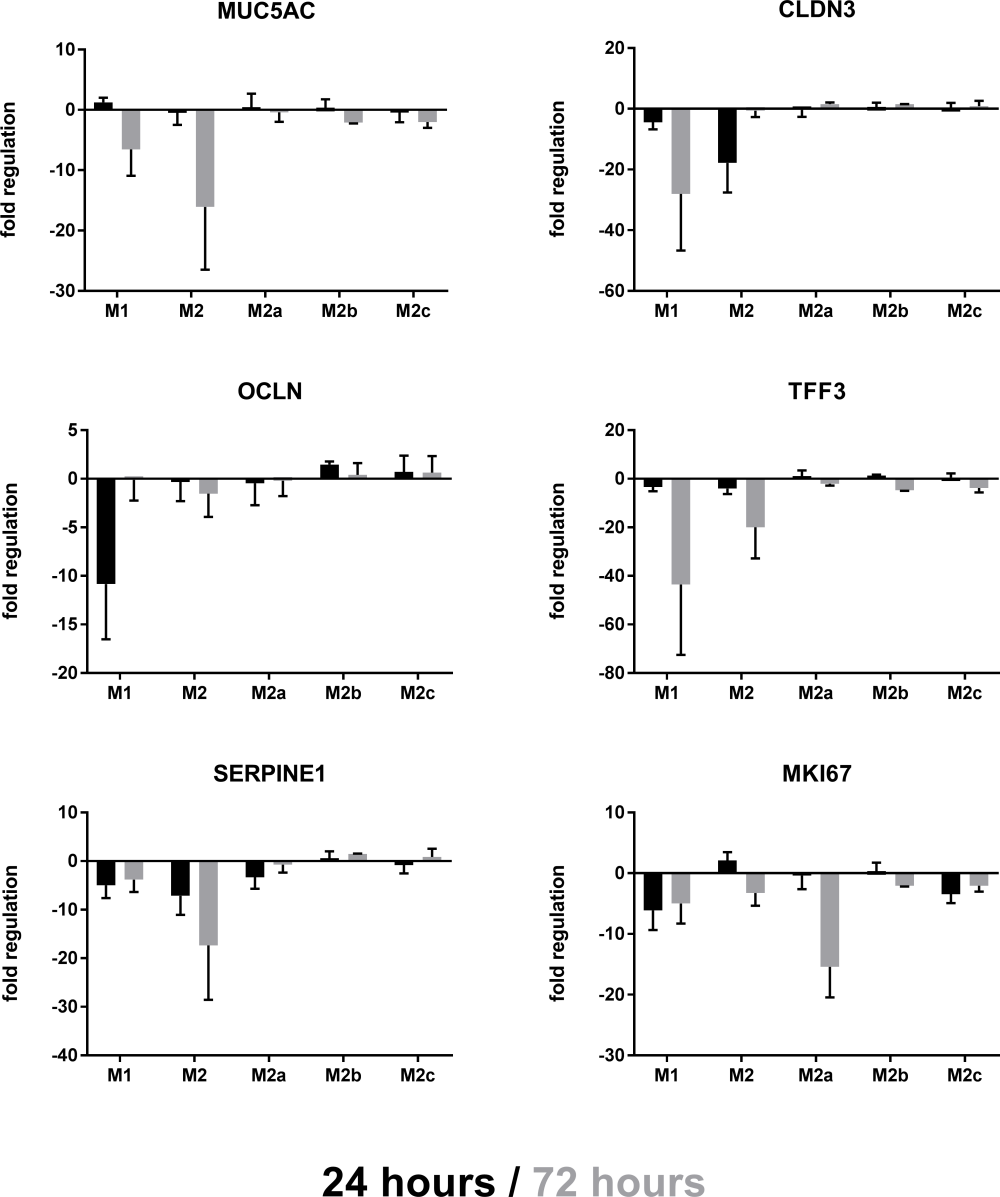
mRNA regulation in the co-cultures. mRNA expression levels in lysates of the co-culture after 24h and 72h for MUC5AC, CLDN3, OCLDN, TFF3, SERPINE1 and MKI67/Ki67; Data are displayed as fold regulation compared to control medium containing macrophage maturation factors; Data is expressed as mean ± SD; n = 3

Following injury, epithelial cells co-cultured with “M_2a_”, “M_2b_” and “M_2c_” macrophages appeared to be less de-differentiated as evidenced by the lack of loss of MUC5AC, CLDN3, OCLN and TFF3 (Fig 4). There was also a reduced loss of SERPINE1 suggesting that the urokinase-mediated matrix degradation system was not impaired. Interestingly, there was a significant reduction in epithelial cell proliferation, as measured by Ki67 gene expression, following injury and co-culture with “M_2a_” macrophages, but not with “M_2b_” or “M_2c_” (Fig 4).

**Fig 5:**
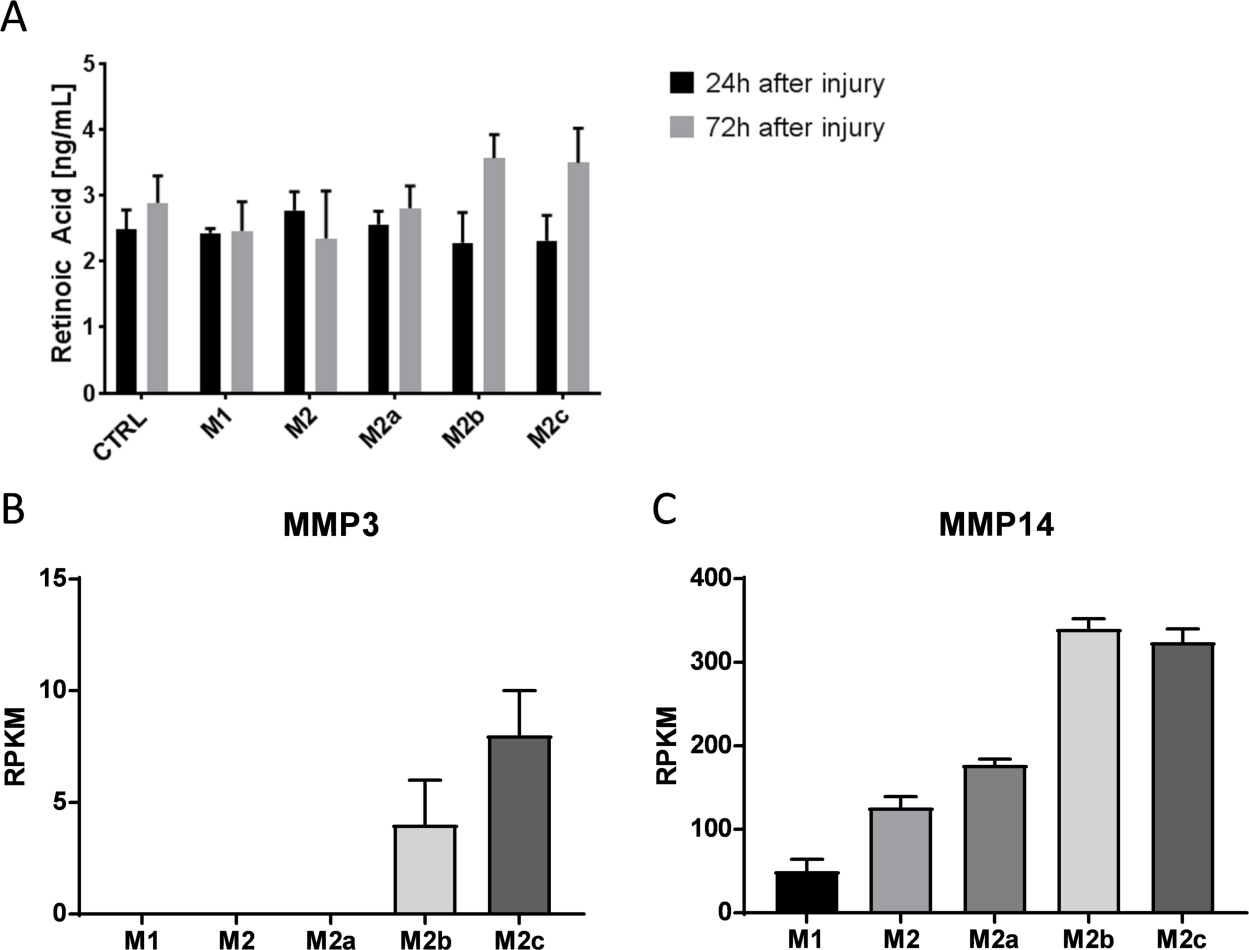
**Retinoid acid amount and MMP3/MMP14 expression. A** Retinoic acid concentration in supernatants of different macrophage subtypes was measured by ELISA **B** MMP3 and **C** MMP14 expression in different macrophage subtypes was measured by TaqMan RPKM = Reads per kilobase per million mapped reads; Data is expressed as mean ± SD; n = 3

In order to understand the effects of the macrophage sub-types on epithelial response to injury, we looked for factors known to modulate fibronectin expression and/or cross-linking/degradation. The “M_2b_” and “M_2c_” macrophages secreted more retinoic acid, compared with “M_1_”, “M_2_” and “M_2a_” into the medium (Fig 5A). Furthermore, “M_2b_” and “M_2c_” macrophages were the only subtypes that expressed MMP3 (Fig 5B) and expressed significantly higher levels of MMP14, compared with “M_1_”, “M_2_” and “M_2a_” sub-types (Fig 5C).

## Discussion

In this study we used primary human small airway epithelial cells differentiated in air-liquid interface culture as a model of the pulmonary epithelium. These cells form a bilayer consisting of basal cells below and differentiated apical cell layer that consists of ciliated columnar epithelial cells and secretory cells. Manual scratching of differentiated small airway epithelial cells results in a reproducible cell-free area that features disrupted cells at the wound edges, which is repaired in a consistent manner by neighbouring cells. Real-time monitoring of epithelial wound repair, revealed a highly migratory process. Cells in the vicinity of the scratch were observed to migrate into the space, a motion that seems to start from several “hot spot” origins, which is consistent with the observations of Erjefält *et al*. [4]. Even after the gap was apparently physically closed, approximately 30 hours after injury, cell movement was still apparent. This was consistent with the recovery of TEER, which was not complete until approximately 48 hours after injury, presumably a measure of the recovery of epithelial integrity and the generation of new tight junctions, despite the fact that the expression of both OCLN and CLDN3 were still down-regulated 72 hours after injury (although mRNA levels as a labile precursor do not necessarily reflect protein levels). Interestingly, the basal cells did not stain positively for the proliferation marker Ki67, but the proliferating cells appeared to reside in the apical layer behind the leading edge of the wound, and only at later time points (the signal was highest 48 hours after injury). This was confirmed by Ki67 mRNA levels that were elevated 48 and even more so at 72 hours post-injury. Furthermore, basal cells were initially observed on the leading edge of the wound at early time points (up to 6 hours), but not thereafter. This would indicate that the initial physical wound closure and recovery of epithelial barrier integrity is predominantly facilitated by migration of differentiated cells from the apical layer into the damaged area, and once barrier integrity is restored, distal apical cells behind the wound proliferate to replenish cell numbers. This observation is contrary to reports that suggest that basal cells play a more dominant role in barrier recovery following oxidative [12, 13] or chemical [14, 15] damage. It could be speculated that this apparent discrepancy is in response to different types of injury and indeed small animal models of mechanical epithelial injury appear to indicate that an initial key event is the cells bordering the lesion to dedifferentiate and flatten which then appear to migrate inwards and over the denuded area to restore the barrier function of the epithelium [16–22]. Interestingly, an intact collagen-coated membrane appears essential for the initial migratory process, although cells appear to be able to eventually cross a damaged membrane as cells on the opposing wound edge become closer, suggesting perhaps a soluble mediator may be responsible. It has been reported that cell motility can be regulated at least in part by Focal Adhesion Kinase [23]. In the immunocytochemistry, FAK was up-regulated on the leading edge of the wound 24 hours after injury which is consistent with the motility observed in the time-lapse videos. We also observed de-differentiation in the apical cells following injury, as described by the down-regulation of FOXJ1 (ciliated cell marker), SCGB1A1 (club cell marker), TFF3 (columnar epithelial cell marker) and ACTA2 (smooth muscle actin), which is also consistent with the observations reported in the small animal models. We also observed a concomitant increase in both mRNA and protein levels of fibronectin following injury. Initially this was apparently intracellular on the leading edge of the wound, but later also apparently deposited extracellularly. Generally, accumulation of extracellular matrix proteins such as collagen or fibronectin is a physiological response to injury [24]. Both soluble and insoluble fibronectin have been shown to increase alveolar epithelial wound healing in an *in vitro* model, increasing cell motility and spreading, but not apparently cellular proliferation [25].

Next we wanted to assess the effect of differentiated macrophage sub-types on the epithelial wound-repair process. Despite numerous attempts, it was not technically possible to determine whether different macrophage per se, or different sub-types affected the rate of wound closure. Nevertheless, there was clear impact of macrophages on some of the epithelial phenotypic characteristics during wound healing. M_1_, but not M_2_, macrophages enhanced the expression of FAK. FAK is reported to be not only associated with enhanced motility, but also epithelial-mesenchymal transition [23], an event linked with COPD [1] and IPF [2]. This is somewhat contradictory to the dogma of M_2_ like macrophages possessing a more pro-fibrotic phenotype [26]. It has been reported that TNF-α induces cytoskeletal rearrangement and FAK activation in endothelial cells via a RhoA-dependent process [27] and that Rho GTPase mediates this permeability [28]. We found that M_1_ macrophages secreted more TNF-α, compared with M_2_, M_2a_, M_2b_ and M_2c_ macrophages (S1 Fig) and that TNFα also mediated increases in epithelial permeability (S2 Fig), suggesting that the increases in FAK observed may be a consequence of activating the TNF-α pathway. Interestingly, M_1_ macrophages were also associated with a greater down-regulation of epithelial Muc5AC, which has also been demonstrated to be down-regulated during EMT [29], but down-regulation was even more pronounced in the co-culture with M_2_ macrophages. Both M_1_, and to a lesser extent M_2_ macrophages, were associated with loss of tight junction protein expression (CLDN3 and OCLN) and the columnar epithelial cell marker TFF3 suggesting promotion of epithelial de-differentiation. SERPINE1 expression was up-regulated following injury, but down-regulated in the presence of both M_1_ and M_2_ macrophages. The protein product of SERPINE1, plasminogen activator inhibitor-1 (PAI-1) has been shown to inhibit MMP activity and therefore it is conceivable that down-regulation of SERPINE1 may promote the deposition of extracellular matrix [30].

Interestingly, the reduction in epithelial CLDN3, OCLN and TFF3 when cultured with M_1_ and M_2_ macrophages was not observed when co-cultured with M_2a_, M_2b_ or M_2c_ macrophages, suggesting that these sub-types may rescue the de-differentiation. It was noteworthy that co-culture with M_2a_, but not M_2b_ or M_2c_ macrophages, was associated with a marked decrease in Ki67 expression, together with reductions in fibronectin expression and soluble fibronectin in the cellular supernatants. Retinoic acid has been described to inhibit both fibronectin expression [31] and cellular proliferation [32] and has been shown to up-regulate MMP3 [33] and MMP14 [34] expression. Interestingly, of the three M_2_ macrophage sub-types M_2b_ and M_2c_ macrophages released the most retinoic acid and expressed the highest levels of MMP-14 and MMP-3, the major fibronectin-degrading MMPs at physiological and acidic pH respectively [35]. Intracellular fibronectin was observed in epithelial cells on the leading edge of the wound after 24 hours when co-cultured with M_2b_ and M_2c_ macrophages, but was not observable in the intracellular, extracellular or soluble spaces after 72 hours. It could be speculated that rather than inhibit fibronectin expression, retinoic acid prevents integration of fibronectin into the extracellular matrix and that the intracellular fibronectin observed at 24 hours in the M_2b_ and M_2c_ co-cultures was subsequently degraded by MMP activity either at neutral pH or in the acidic environment found in the vicinity of activated macrophages [36].

In conclusion, we have generated an *in vitro* mechanical injury model in air-liquid interface differentiated primary human small airway epithelial cells that recapitulates many of the characteristics observed during epithelial wound injury in both human tissue and small animal models. Wound closure was initially associated with de-differentiation of the differentiated apical cells and rapid migration into the wound site, followed by proliferation of apical cells behind the wound edge, together with increases in FAK expression, fibronectin and reduction in PAI-1 which were associated with cell motility and extracellular matrix deposition. M_1_ macrophages promoted FAK expression and both M_1_ and M_2_ macrophages promoted epithelial de-differentiation. Collectively these data highlight the complex nature of epithelial wound closure, the differential impact of macrophage sub-types on this process, and the heterogeneic and non-delineated function of these macrophages.

## Materials & methods

### Small airway epithelial cells

Clonetics™ Cells isolated from the 1 mm bronchiole area of normal human lung tissue (Lonza, donor #408031, 57 year old female; Caucasian; passage 2; seeding efficiency: 85%; doubling time: 37 h; viability: 79%) were cultured and differentiated according to the manufacturer’s instructions. As stated by Lonza, the cells were isolated from donated human tissue after obtaining permission for their use in research applications by informed consent or legal authorization. Briefly, after thawing, SAECs were seeded into a cell culture flask (day -8) in Clonetics S-ALI growth medium. On day -4, cells were trypsinised and seeded on transwell inserts (Corning, #3470) in 24 well plates at a density of 22x10^3^ cells/well. Airlift of cells was performed on day 0 by removing apical medium. Basolateral growth medium was substituted with differentiation medium (Clonetics S-ALI differentiation medium). Remaining growth factors on the apical side were removed by washing with prewarmed PBS. SAECs were differentiated for >4 weeks in an ALI until a pseudostratified epithelium, including mucus production and beating cilia, was observable under the microscope.

### Human monocyte derived macrophages

Peripheral blood mononuclear cells (PBMCs) were isolated from healthy human whole blood by means of density gradient centrifugation using Ficoll-Paque™ and a Leucosep Tube (Greiner Bio-One GmbH) according to manufacturer’s instructions. Blood was donated by internal donors at the centre for occupational health at Boehringer Ingelheim in Biberach, the donors provided signed informed consent that allows use for scientific purposes. CD14 positive monocyte purification was performed by magnetic activated cell sorting (MACS) according to the manufacturer’s instructions (Monocyte Isolation Kit II, Miltenyi Biotec) and seeded 1.5x10^5^ cells/cm^2^ in XVIVO-10 medium (Lonza) into Thermo Scientific Nunc UpCell Surface cell culture plates. Medium was supplemented with either 100 ng/mL Granulocyte-Macrophage Colony Stimulating Factor (GM-CSF) to induce an M_1_ phenotype or Macrophage Colony Stimulating Factor (M-CSF) to induce an M_2_ phenotype for 7 days. On day 7, differentiated M_2_ macrophages were additionally stimulated with maturation factors for a further 3 days to induce sub-phenotype differentiation; either 20 ng/mL IL-4 and 20 ng/mL IL-13 (M_2a_), 20 ng/mL LPS and 100 μg/mL immune complex (M_2b_) or 20 ng/mL IL-10 (M_2c_). Immune complexes were made by combining purified Beriglobin® (Intravenous immunoglobulin G (IVIG) preparation, CSL Behring) and Twinrix® (Hepatitis A/B vaccine preparation, GlaxoSmith Kline) with subsequent purification by means of size exclusion chromatography. Differentiated macrophages were detached from the UpCell Surface cell culture plates by letting the plates cool down to room temperature and cell concentration and viability was determined using the Countess™ Automated Cell Counter (Invitrogen).

### Scratch assay and live cell imaging

Injury simulation in differentiated SAECs was performed by manually scratching once across the cell layer on the transwell using a sterile 200 μL pipette tip (Eppendorf) taking care not to damage the membrane or to remove the collagen coating on the membrane. This would negatively influence the wound healing process. The size of the initial scratch area and subsequent cellular regrowth into the injured site was monitored using a CellIQ Analyser (Chip-Man Technologies Ltd.). Wound healing of scratched SAECs was monitored using Live Cell Imaging. Cells were cultivated at 37°C with a gas flow of 14 mL per minute. Gas flow was repeatedly turned on for 15 minutes followed by 30 minutes without gas. Humidity was not regulated automatically by the system, but was achieved by filling residual wells and intermediate spaces of the 24 well plate with PBS. Cells were monitored for three days without medium exchange. To improve the visual contrast between cells and membrane pores, cells were labelled with 25 μM 5-(and-6)-carboxyfluorescein diacetate, succinimidyl ester (CFSE; C1157, Thermo Fisher Scientific) for 20 minutes at 37°C immediately after injury. Phase contrast images were captured continuously and at every fourth cycle, the GFP channel was turned on to capture fluorescent images. Single images were stitched to grids and merged to videos.

### Macrophage-epithelial cell co-culture

To model the interaction between alveolar macrophages and airway epithelial cells, a co-culture system was developed. SAECs were cultivated as described above. Epithelial cells were scratched manually and the differentiated macrophages were added apically (5x10^5^ cells per transwell). X-VIVO 10 medium (Lonza), which was supplemented with different stimulation factors, was added apically to promote macrophage differentiation into different phenotypes. Apical addition of the macrophage differentiation factors did not alter epithelial cell viability, growth or gene expression (data not shown).

### Transepithelial electrical resistance (TEER)

Transwells of ALI cultures were placed into a cellZscope (nanoAnalytics) for TEER measurement. Medium was added basolaterally (700 μL) and apically (200 μL) to enable impedance measurement. To maintain optimal culture conditions, the cellZscope was placed in a tissue culture incubator (37°C / 5% CO_2_). TEER was measured continually for up to 3 days.

### Immunofluorescent imaging

SAECs were fixed in 300 μL ice cold (1:1) Acetone-Methanol for 15 minutes. Cells were washed three times with 0.1% BSA in PBS and blocked with 5% BSA in PBS for at least one hour. The primary antibodies in 0.1% BSA in PBS were incubated on the cells for one hour, washed and secondary antibodies were incubated for one hour at 37°C in the dark and washed once again. The transwell membrane was punched out with a biopsy punch, embedded with ProLong® Gold Antifade Mountant with DAPI (Thermo Fisher Scientific, P36935), placed on a glass slide, covered with a coverslip and examined under a confocal microscope (Zeiss Laser Scanning Microscope (LSM) 710). The following antibodies were used: Cytokeratin 5 Rabbit Monoclonal Antibody (EP1601Y; NB110-56916, Novus; 1:200); MUC5AC Mouse Monoclonal Antibody (45M1; MA5-12178, Thermo Fisher Scientific; 1:20); Ki-67 Mouse Monoclonal Antibody (180192Z, Thermo Fisher Scientific; 1:100); Fibronectin Mouse Monoclonal Antibody (3F12; MA5-14737, Thermo Fisher Scientific; 1:40); Vimentin Rabbit Polyclonal Antibody (S82; AP2739a, abgent; 1:400); FAK/PTK2 Rabbit Monoclonal Antibody (5H18L19; 701094, Thermo Fisher Scientific; 1:13); Smooth Muscle Actin Mouse Monoclonal Antibody (MA5-11547, Thermo Fisher Scientific; 1:400); Goat anti-Mouse IgG (H+L) Secondary Antibody, Alexa Fluor® 647 conjugate (1:400); and Donkey anti-Rabbit IgG (H+L) Secondary Antibody, Alexa Fluor® 568 conjugate (1:400). F-actin was stained with ActinGreen™ 488 ReadyProbes® Reagent (Thermo Fisher Scientific, R37110).

### Quantitative PCR

Cells were lysed with 200 μL buffer RLT (lysis buffer) for 10 minutes and frozen at -20°C. RNA was isolated using the RNeasy Mini Kit by QIAGEN according to manufacturer’s instructions. RNA concentration was determined using a NanoDrop 8000 spectrophotometer (Thermo Fisher Scientific). Isolated RNA (0.8μg) was reverse transcribed into cDNA using the High-Capacity cDNA Archive Kit (Applied Biosystems) in a RT-PCR cycler (Biometra TGradient Thermocycler) at 25°C for 10 minutes, 37°C for 120 minutes and 85°C for 5 minutes. QuantiFast® Probe PCR + ROX Vial kit (QIAGEN) was used to prepare samples. Quantitative PCR was performed using dual-labeled probes (TaqMan® probes) with the Applied Biosystems ViiA™ 7 real-time cycler using the following probe sets (all Thermo Fisher Scientific): 18S rRNA (4319413E), RNA polymerase II (Hs00172187_m1), Cytokeratin 5 (Hs00361185_m1), Mucin 5AC (Hs00873651_mH), Ki67 (Hs01032443_m1), Fibronectin 1 (Hs00365052_m1), Vimentin (Hs00185584_m1), Claudin 3 (Hs00265816_s1), Claudin 9 (Hs00253134_s1), Occludin (Hs00170162_m1), Forkhead Box J1 (FoxJ1; Hs00230964_m1), Secretoglobin Family 1A Member 1 (SCGB1A1; Hs00171092_m1), Trefoil Factor 3 (TFF3; Hs00902278_m1), Plasminogen Activator Inhibitor (SERPINE 1; Hs01126606_m1), Smooth Muscle Actin (Hs00426835_g1), Plasminogen Activator, Urokinase (Hs01547054_m1), and Urokinase Receptor (Hs00958880_m1).

### ELISA

Soluble human fibronectin was detected using a kit supplied by affymetrix eBiosciences (Human Fibronectin Platinum ELISA) and retinoic acid was measured using a kit supplied by cusabio (Human retinoic acid ELISA kit), both according to manufacturer’s instructions. Absorbance was read on a spectrophotometer using 450 nm as the primary wavelength and 620 nm as reference wavelength (Spectramax M5e; Molecular Devices).

## Supporting information

S1 Fig TNFα production by different macrophage subtypes measured via MSD. (TIF)

S2 Fig TEER development of SAEC treated with 10 ng/mL TNF-α (orange line) or plain medium (black line) over 64h. (TIF)

S1 Video Wound healing response along the wound edge. (WMV)

S2 Video Influence of the collagen coating on the wound closure. (WMV)
