# Supplementary figures and images for: Opposing effects of in vitro differentiated macrophages sub-type on epithelial wound healing
Source: PLoS One. 2017 Sep 1;12(9):e0184386. doi: 10.1371/journal.pone.0184386 (PMC5581193; doi:10.1371/journal.pone.0184386)

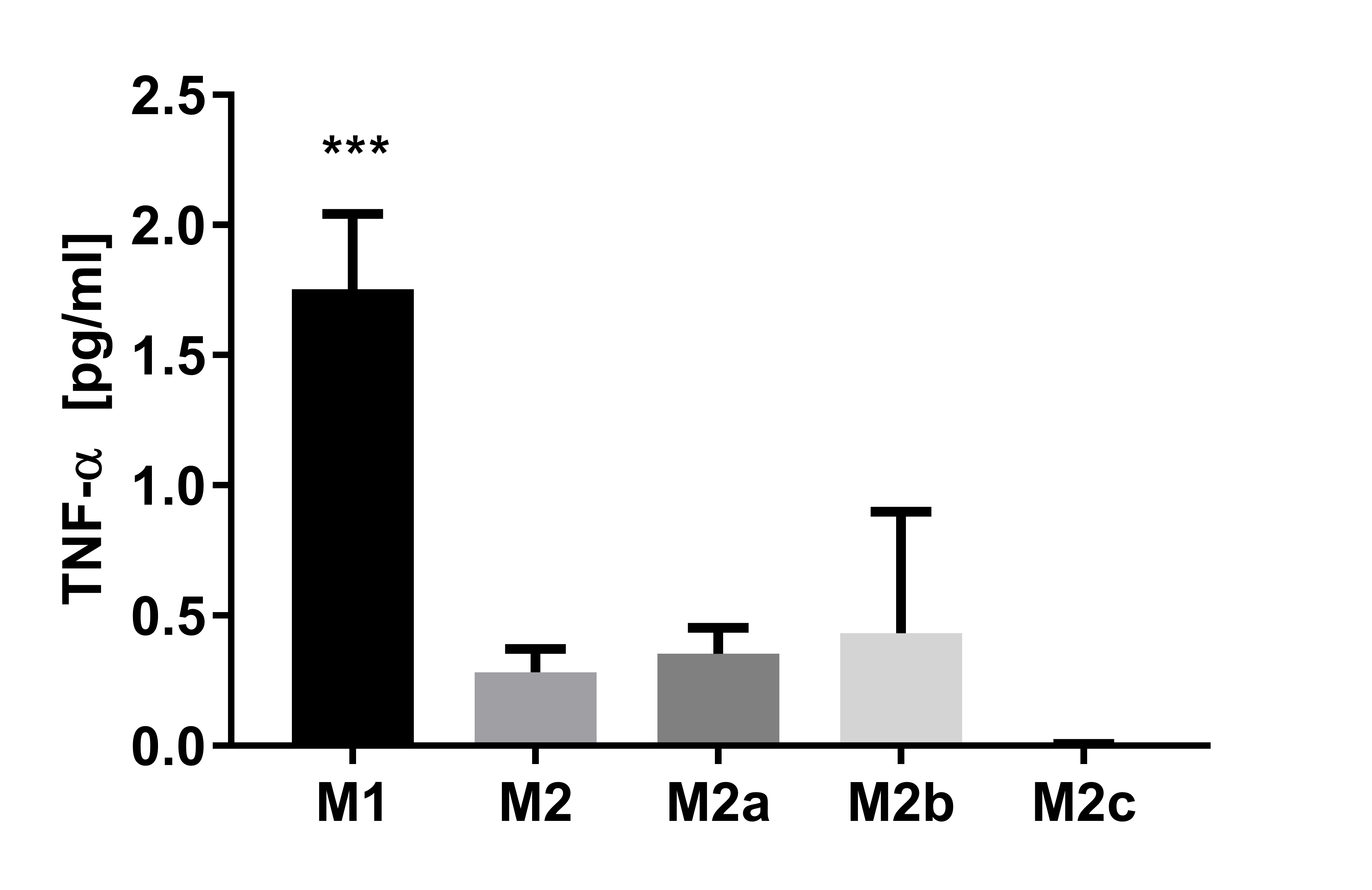

Supplement: S1 Fig — (TIF) [file pone.0184386.s001.tif]

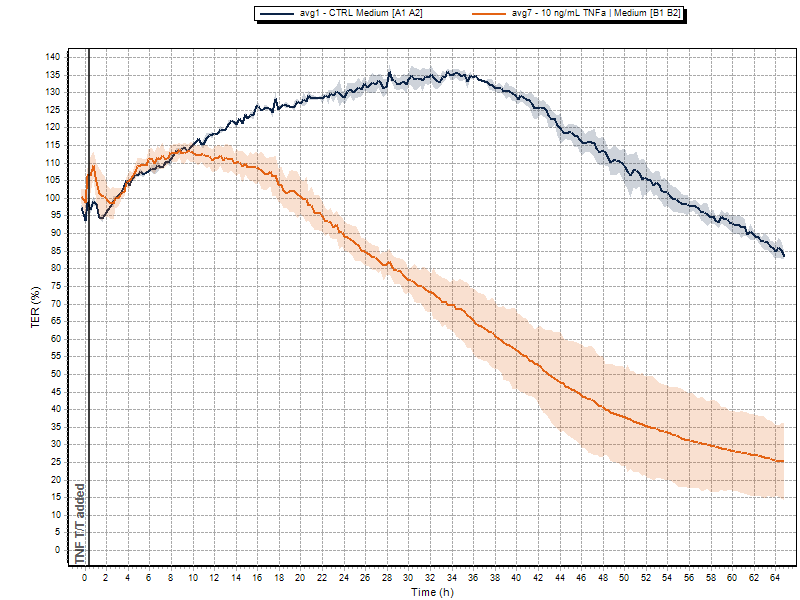

Supplement: S2 Fig — (TIF) [file pone.0184386.s002.tif]
